# Surviving a transfixing gunshot wound to the head 70 years ago

**DOI:** 10.1007/s12024-018-0025-4

**Published:** 2018-10-20

**Authors:** Patricia Shirley de Almeida Prado, Sergio Ricardo Marques, Yara Vieira Lemos, Eugénia Cunha

**Affiliations:** 1 Department of Biomorphology, Federal University of Bahia, Salvador, BA 40110-902 Brazil; 2 Institute of Medicine & Biomedical Education, St. George’s University of London, London, SW17 0RE UK; 3 Department of Morphology and Genetic, Discipline of Topographic and Descriptive Anatomy, Federal University of São Paulo, São Paulo, SP 04023-900 Brazil; 4 Laboratory of Forensic Anthropology, Legal Medicine Institute, Civil Police, Belo Horizonte, MG 31630-903 Brazil; 5 Faculty of Medical Sciences of Minas Gerais, Belo Horizonte, MG 30130-110 Brazil; 6 Laboratory of Forensic Anthropology, Centre for Functional Ecology, Department of Life Sciences, University of Coimbra, 3000456 Coimbra, Portugal; 7 National Institute of Legal Medicine and Forensic Sciences, Largo da Sé Nova, 3000-213 Coimbra, Portugal

**Keywords:** Survival, Gunshot wound, Forensic anthropology, Cranium, Healed fracture, Skull collection

## Abstract

Surviving a gunshot wound to the head is a rare event, particularly in the past when medical treatment was much less advanced than it is today. Moreover, the finding of such a case as an identified specimen within a museum collection is even more uncommon. This led us to report on this unique case in this paper as it poses a challenge to forensic anthropology and provides a unique educational oppourtunity. The skull from the Collection at the Cranium Museum in the Department of Morphology and Genetic at the Federal University of São Paulo (UNIFESP) dates back to 1946. For trauma registration the bone location, severity, trauma aetiology, trauma classification, description, callus formation, periosteal reaction, degree and success of repair, and an estimate of the time elapsed since the trauma, were all assessed. To explore the case radiologically a CT scan of the skull was performed. Considering the survival of the patient and the morphology of the wound it is likely that the injury was caused by a small calibre, low-velocity gunshot. The bullet path shows an almost vertical direction on the right side of the individual’s splanchno and neurocranium. The path of the projectile is consistent with a suicide attempt, although the possibility of a homicide simulating a suicide cannot be discarded. This case highlights how informative such cases can be to the practice of forensic anthropology.

## Case report

### Case provenance and biological profile

During the collection of data for research purposes in the Brazilian skull collection at the Cranium Museum in the Department of Morphology and Genetic at UNIFESP a rare case was identified. A skull with an impressive and quite uncommon healed wound was discovered. Started in 1930, the skull collection at UNIFESP stands out because of its extensive documentation of mostly contemporary skulls (dates of death: 1933–1970). The majority of the skulls are documented with data such as name, sex, age, ancestry, profession and *causa mortis*.

The skull we investigated for this paper is registered as number 150 in the collection and has the following accompanying data: the initials of the name UO, male black Brazilian, factory worker, single. Date of death: 14/10/1946; age: 42 years, causa mortis: heart failure. Unfortunately we werer not able to locate any medical records associated with this case. Despite having been autopsied, where more information regarding *causa mortis* would have been provided, our search for further information was unsuccessful as we were told that all the reports from before 1950 had been destroyed. Our assessment of the biological profile of the skull supported the recorded sex, age and ancestry. The external aspect of the skull showed no pathological features, although this individual presented with a supra nasal suture, sagittal ossicles and lambdoid ossicles, a patent condylar canal and supra mastoid crest all present on both sides.

### The lesions

The skull displays a healed hole on the anterior-lateral part of the frontal bone with a fragment of metal adherent (Fig. 2e), the lesion which first caught our attention.

To best describe the trauma we followed the recommendations of Cunha and Pinheiro [1]. We tried to register the following features: bone location; severity; trauma aetiology, trauma classification, description, callus formation, periosteal reaction, degree and success of repair, and estimation of time elapsed since the trauma. Furthermore, we also took into account the antemortem signs endorsed by the scientific working group for forensic anthropology [2].

To explore the case radiologically a CT scan of the skull was performed with the following protocol and details. This skull was CT-scanned with a Philips Brilliance 64 CT Scanner at the Federal University of São Paulo (Hospital São Paulo). The CT series for the sample was recorded using a tube voltage of 120 kV, slice thickness of 2 mm, and slice increment of 1 mm, 64 × 0.625-mm collimation, matrix 512 × 512 were made in the coronal (transverse) and sagittal plane. Visualization of the skull CT scan was produced using the software application MicroDicom viewer (MicroDicom viewer, version 2.7.9).

### Macroscopic analysis

While a thorough macroscopic analysis was carried out, another bone orifice was noticed on the right palatine. When we tried to link the two orifices, the hypothesis that the decedent havd survived a gun shot wound was the most probable. We suggest that the entrance wound was the one located in the oral cavity and the exit wound was the one located on the frontal bone. The reasons for this are provided below.

**Fig. 1:**
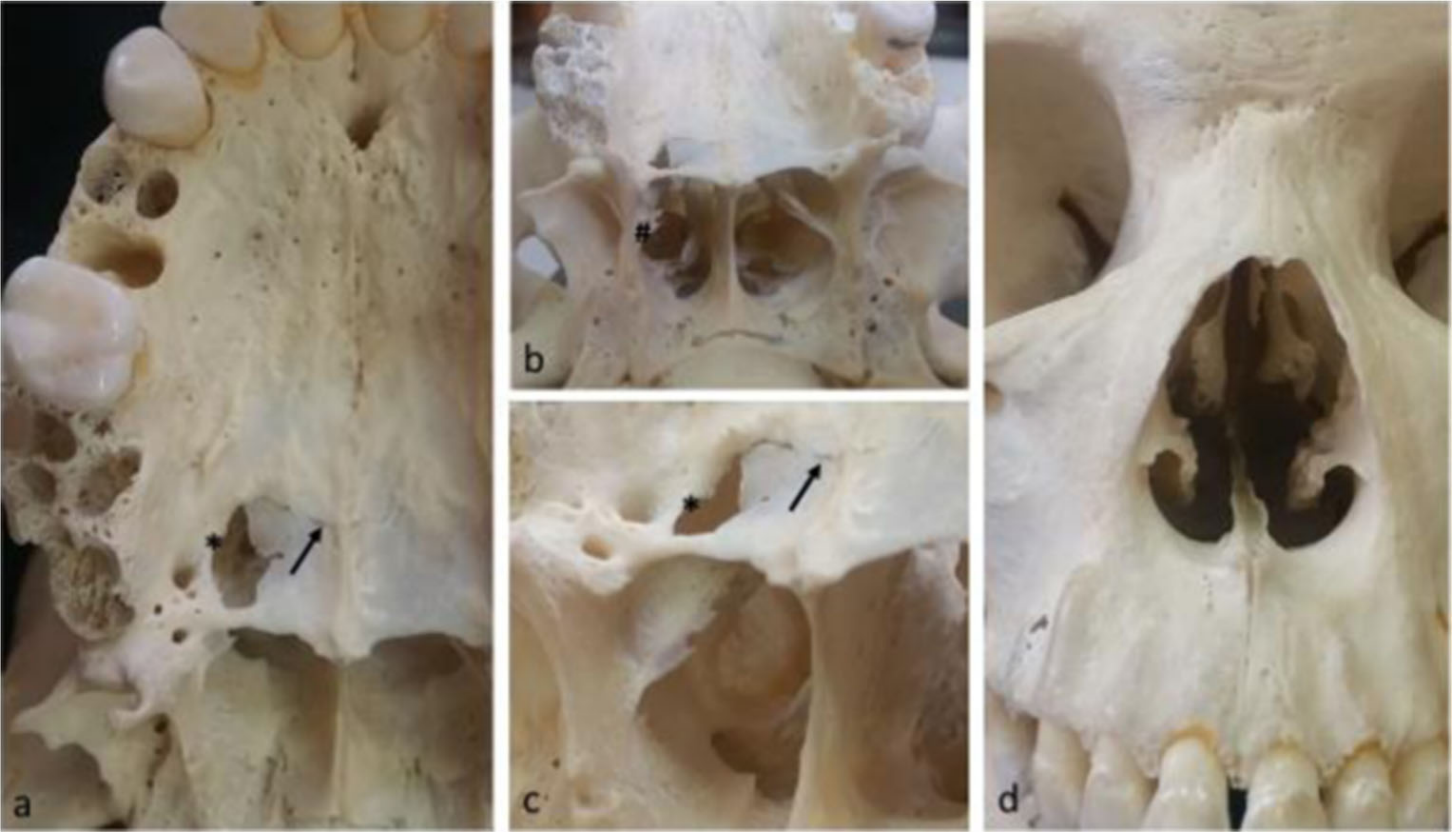
Frontal and basal cranial views. 1**a**, **b** and **c**: palatine view of the GSW entrance orifice. Note the smooth round appearance of the healed trauma (*); the arrow shows the healing process of the fractured transverse palatine suture (bony bridges). 1**d**: frontal view of nasal cavity showing no bony damage to the anterior portion of this cavity

**Fig. 2:**
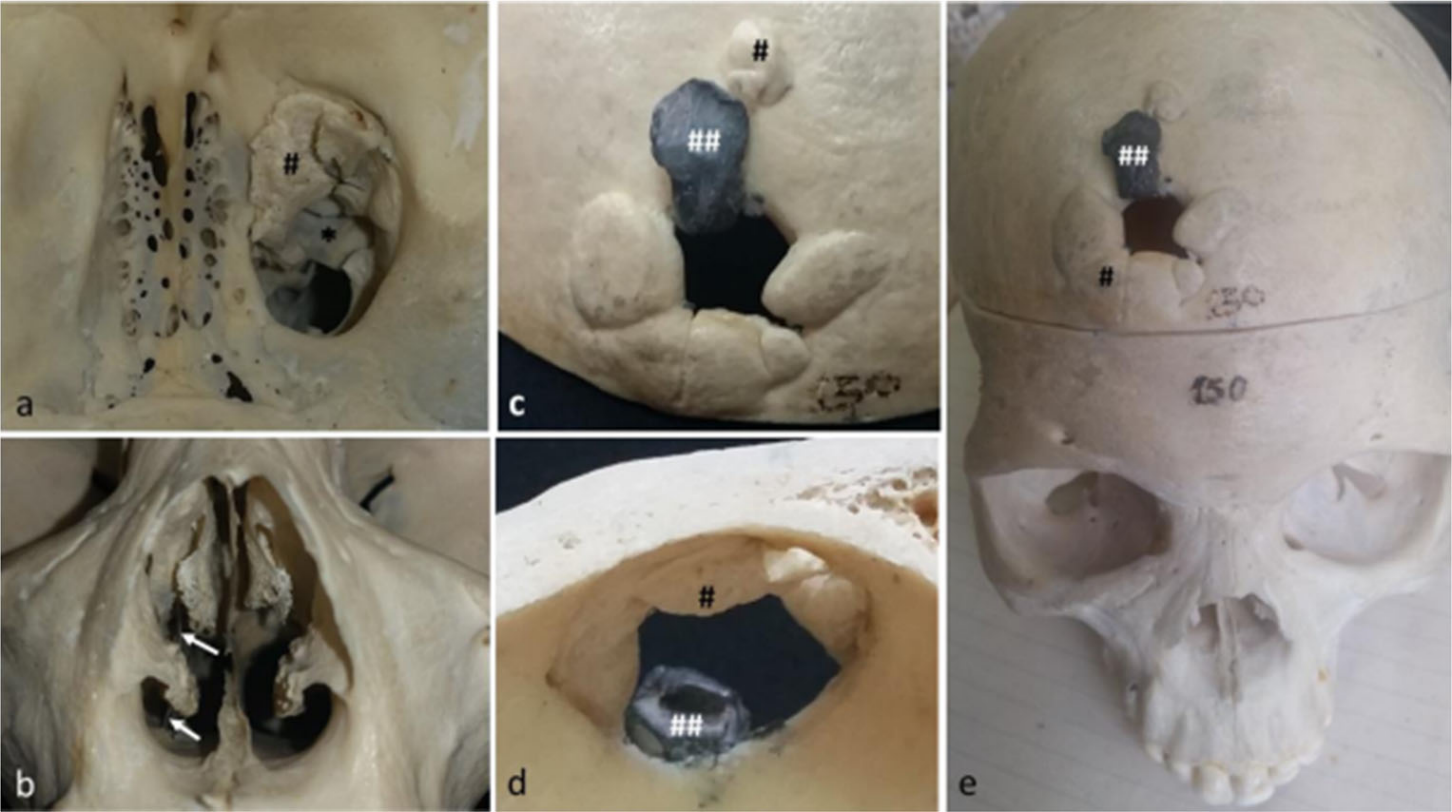
External and internal view of the cranial vault and frontal view of nasal cavity. 2**a**: superior view of the anterior cranial fossa showing on the right side the ossified bullet pathway (*) and the ossified bone fragments on this path, ethmoidal cells labyrinth level. 2**b**: anterior view of the nasal cavity, note the bullet pathway through a metallic device (arrows). 2**c**, **d** and **e**: external (**c**, **e**) and internal (**d**) cranial vault showing the exit GSW. Note the metallic bullet fragment (##) firmly adhered to the external cranial vault on the exit wound as well as the bony fragments (#)

The hole in the oral cavity displays evidence of internal bending and no bevelling. The wound has a round appearance on the right side of oral cavity between posterior portion of the maxillary palatine process and horizontal plate of palatine bone (Fig. 1a–c). The lateral margin of this injury shows an osteogenic reaction leading the classification of this injury as antemortem (Fig. 1a and b*), a healing fracture line following the palatomaxillary suture is also evident (Fig. 1a–c). Because of these features, it was most probably the entrance wound. The bullet path is evident in an almost vertical direction on the right side of the individual’s splanchno and neurocranium (Fig. 2b). Furthermore, after crossing the palatine vault, the bullet went through the nasal cavity in the lateral-posterior position, leaving a clear bullet shape hole in this cavity (Fig. 2a). It is also possible to observe both antemortem and post-mortem fractures on the posterior portion of the middle and inferior nasal conchae (Fig. 1b#). Otherwise, the anterior view of the nasal cavity shows no modification related to this trauma (Fig. 1d).

Furthermore, the projectile went through the splanchnocranium and reached the anterior cerebral fossae alongside the cribriform plate, into the ethmoid labyrinth (Fig. 2a*) without affecting the orbital wall and cavity. At this level ossified bone fragments on the projectile path (Fig. 2a#) can be seen. The external surface of the cranial vault shows several bone fragments adhered and ossified to the margin of the exit wound (Figs. 2c–e#) the most impressive feature being a metallic piece of debris trapped on the external cranial surface (Fig. 2c–e##).

**Fig. 3:**
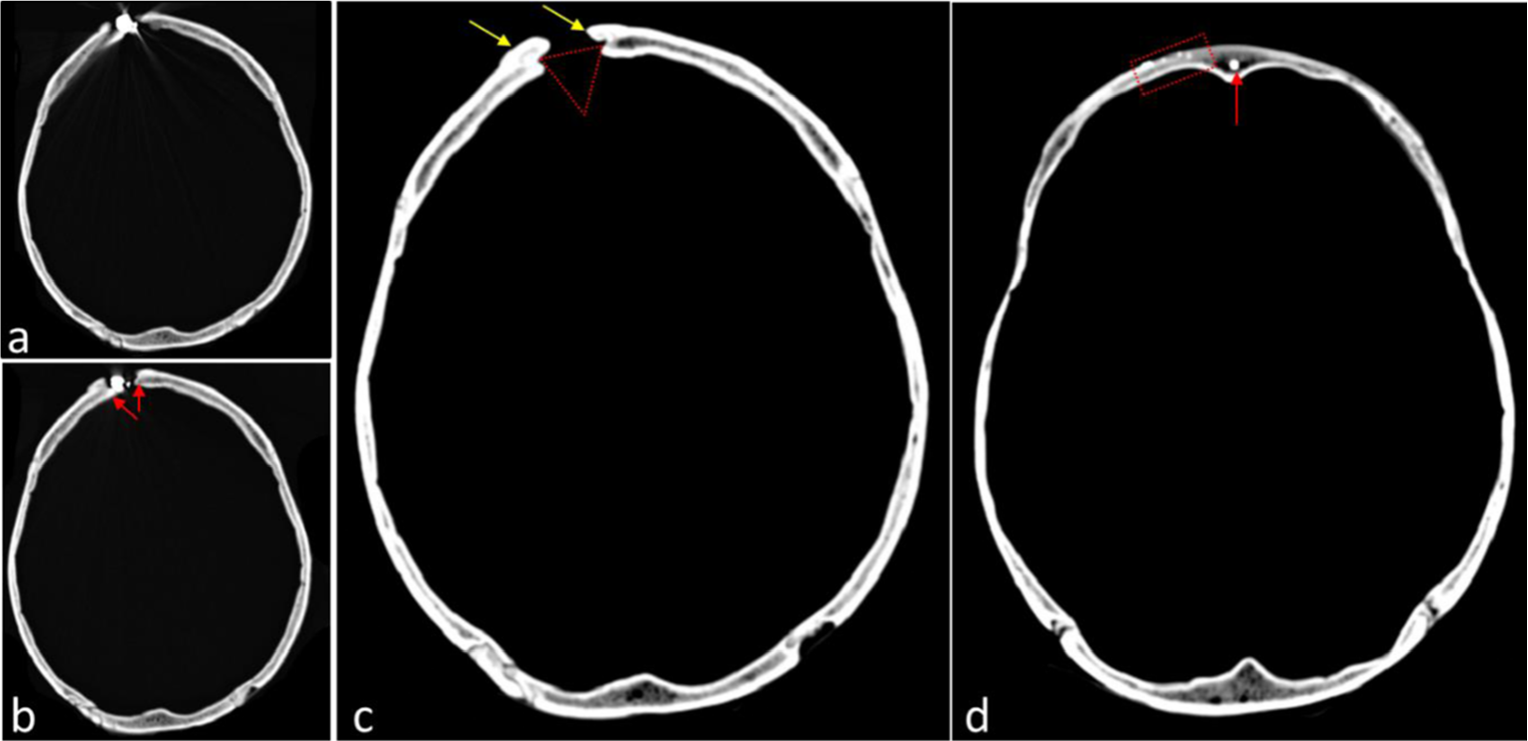
Axial CT images of the cranial vault. 3**a** and **b**: image of high radiological density corresponding to the remnants of the firearm projectile (also seen Fig. 2**c**–**e**). In Fig. 3**b** it is also possible to see the image of the Bonet’s funnel (red arrows) and rounded borders, characterizing bone remodelling, evidence of ante mortem healing. 3**c**: image of Bonet’s funnel in the frontal bone (red triangle). The area of bone remodelling was more intense in the external table of the frontal bone (yellow arrows). 3**d**: images of high radiological density at the frontal bone (red rectangle) and in the frontal sinus (red arrow) consistent with metallic dust resulting from the firing of a firearm

**Fig. 4:**
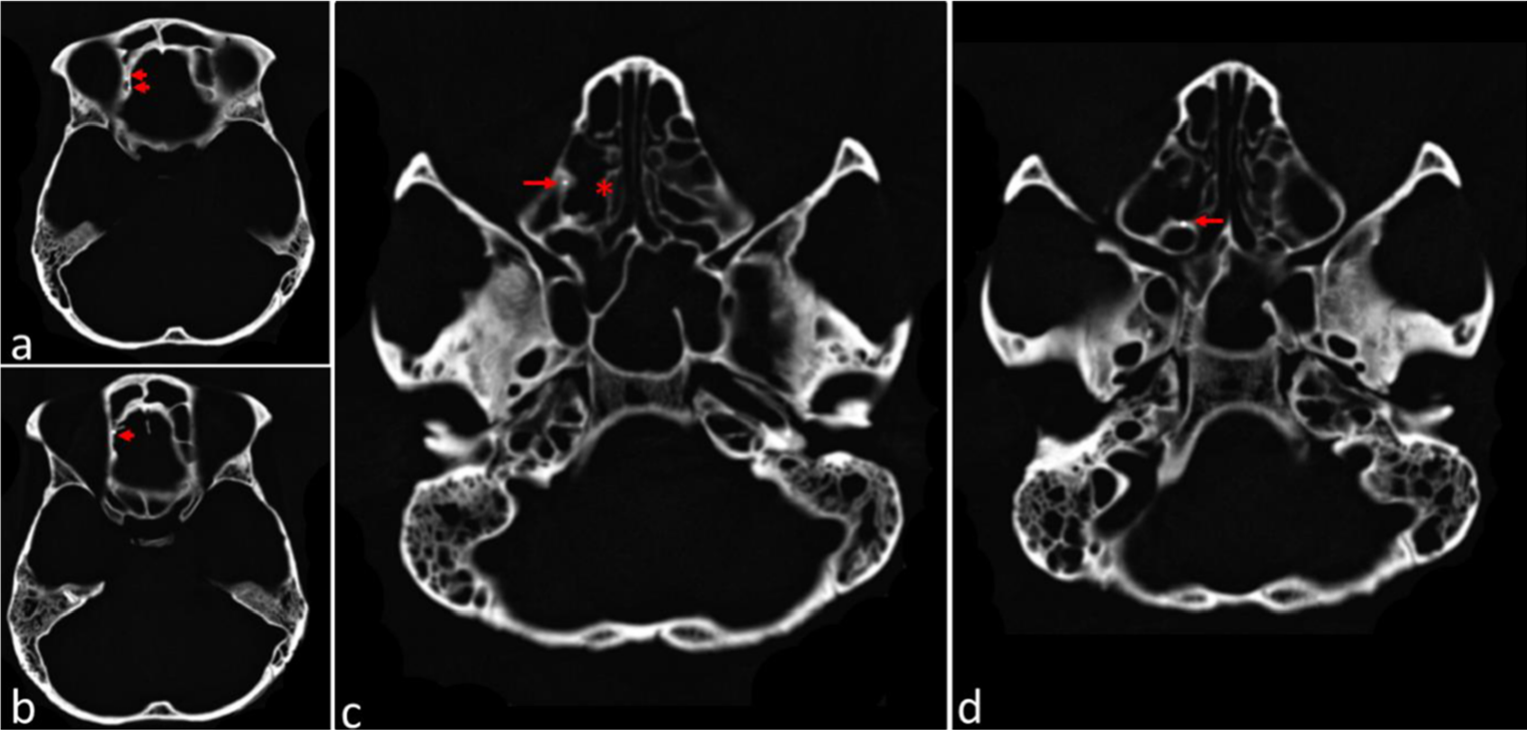
Axial CT images at the level of the ethmoidal labyrinth and orbital cavity. 4**a**: the right orbital blade of ethmoid bone had an area with images of high radiological density (red arrows). 4**b**: at the ethmoidal bone, behind the right side of the frontal sinus, there was an image of high radiological density (red arrow). 4**c** and 4**d**: the bone framework of the right ethmoidal cells had an area with an image of high radiological density (red arrow). The ethmoidal cells on the right (red asterisk), compared to the left, had areas of bone loss, which indicates the path and damage caused by the firearm projectile

**Fig. 5:**
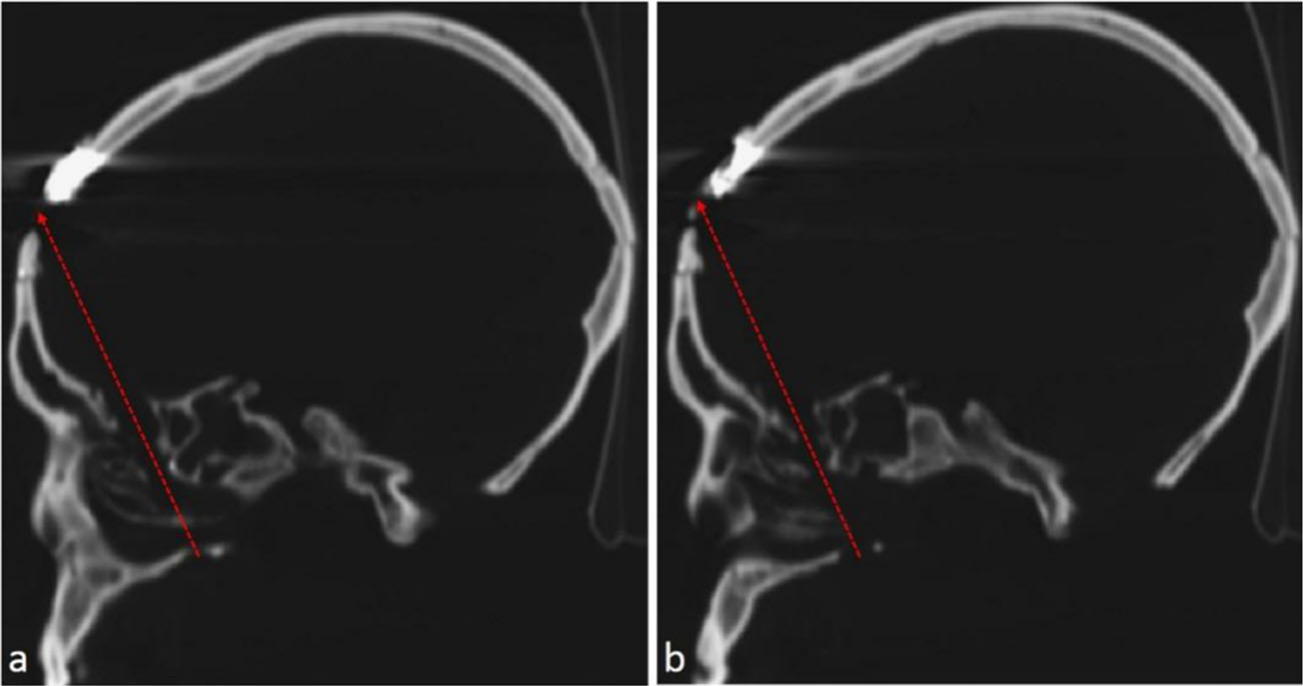
Sagittal CT Images of the right right side on the level of the ethmoidal labyrinth. 5**a** and **b**: drawing of the probable bullet trajectory (red arrow a and b). Note the high radiological density on the frontal bone (posterior to the head of the arrow) corresponding to the remnant of the firearm projectile

Fortunately the CT scan added further information to the case; in several transvere sections there were high dense spots consistent with the presence of metallic fragments of the bullet that resulted as a consequence of the so-called snow storm. Tracking these residues gave clues about the bullet’s path (Figs. 3, 4 and 5), the lowest level where the residues can be seen is on the cribriform plate whereas the highest are on the frontal bone, at the exit wound where most of the bullet remained stuck (Figs. 3 and 4).

The bullet path runs along an uncommon posterior to anterior trajectory (Fig. 5a and b). Macroscopic features such as the rounded entrance wound in the hard palate, the destruction of ethmoidal cells, and the exit wound on the right frontal bone, can also be seen in the CT scan sagittal images (Fig. 5a and b) supporting our understanding of the most probable bullet path.

## Discussion

Traumatic wounds to the head are very common in forensic anthropology. However, since severe injuries to the skull often result in death, it is uncommon to find severe antemortem lesions affecting the cranium. Our case concerns a cranium with a gunshot wound and a rare example of heterotopic ossification in the path of a projectile.

Cunha and Pinheiro [3] note that gunshot wounds, if not lethal, will be observed as holes, maybe smaller than the initial lesions, bordered with smooth margins, occasionally with a thin layer of new bone closing the orifice. The classic appearance of internal bevelling on the entry hole and external bevelling on the exit wound will occasionally be unapparent, depending on the time of the injury. The present case corroborates these characteristics.

It seems that in this case the bullet suffered fragmentation upon entry and it reached the frontal bone with significantly less energy, meaning that it did not exit the skull completely. It should be mentioned that there are no fracture lines, neither radiating nor concentric fractures in the cranial vault. Taking into account that a fracture is created to dissipate the energy, the unharmed mandible, limited trauma, and survival of the individual support the hypothesis that the bullet was a low-velocity missile. It is well known that the mandible, in general, protects the rest of the face in submental gunshot wounds. As the first bone to be affected and broken, most of the kinetic energy is absorbed in a fracture limiting further damage [4]. In this case, the first bony impact of the bullet was the hard palate and cribriform plate that could not absorb a high-velocity bullet.

Examining the skull and topographical sections on the CT images, the estimated route of the projectile inside the head was from posterior to anterior, from caudal to cranial, slightly from left to right. This path is atypical leading to a different hypothesis concerning the incident scenario.

Taking in to consideration that the patient survived, along with the morphology of the wound in the skull and the trapped bullet in the cranial vault, it is likely that a small calibre, low-velocity gunshot was the cause of the damage. The entrance to the palatine roof is atypical when compared to a classic entrance wound because it exhibits bending, a linear fracture line, and signs of remodelling bone, which can be seen in Fig. 1a and c. It was most likely a shot from a revolver or a shotgun, remembering that this case dates back to 1946.

The unusual wound pattern reported in this case contributesto discussion regarding the type of firearm that was used. To reach the trigger of a 76 cm barrel gun placed in the submental area, an individual with a regular arm length must hyperextend the neck and lower their shoulders [4]. This body position generally causes the bullet to affect the lower third of the face without it reaching intracranial structures. The literature suggests that the most optimistic prognosis and survival rates are seen in cases of self-inflicted wounds in the submental and intraoral regions, using low-energy handguns or a rifle [5].

Regarding the manner of death, the trajectory path is consistent with a suicide although the possibility of a homicide simulating a suicide can not be discounted.

This case reinforces and illustrates Ortner’s comment “the extent of injuries to the skull that an individual can survive is often remarkable” [6]. Seventy-five years ago medical treatment was poor, and secondary diagnostic methods such as radiology were also much less developed, which reinforces the uniqueness of this case: survival from a gunshot wound to the head in 1946.

For forensic anthropology, a fundamental question concerning antemortem trauma remains: How long did the individual survive the lesion? [1]. Post-traumatic interval (PTI) is now one of the key research topics in forensic anthropology. The chronology of traumatic injury in the skull is more intricate compared to that of the long bones due to the distinct healing pattern of the skull. Unlike the long bones, which tend to form a callus, skull bones tend to develop bone bridges between the fragments during the healing process [7]. These healing differences can be explained by studies in embryology; the course of fracture healing mirrors the molecular aspects of bone development [8].

Despite the different appearance of skull bones that have undergone repair and healing, the remodelling process in this case was quite clear. However, attempting to determine the chronology of the injuries was one of the main challenges in this case, mainly because of the impossibility of doing histology. Finally, these antemortem lesions could have acted as unique identity factors in a forensic anthropology case as such lesions are strong identifiers. The victim had most probably been to hospital to treat his injuries, making it possible to match the post-mortem examination with the medical files and achieve a positive identification.
